# Supplementary material for: Genomic and Molecular Profiling of Human Papillomavirus Associated Head and Neck Squamous Cell Carcinoma Treated with Immune Checkpoint Blockade Compared to Survival Outcomes
Source: Cancers (Basel). 2021 Dec 16;13(24):6309. doi: 10.3390/cancers13246309 (PMC8699559; doi:10.3390/cancers13246309)
Supplement: Supplementary file 1 [file cancers-13-06309-s001.zip › cancers-1471516-supplementary.pdf]

cancers

MDPI

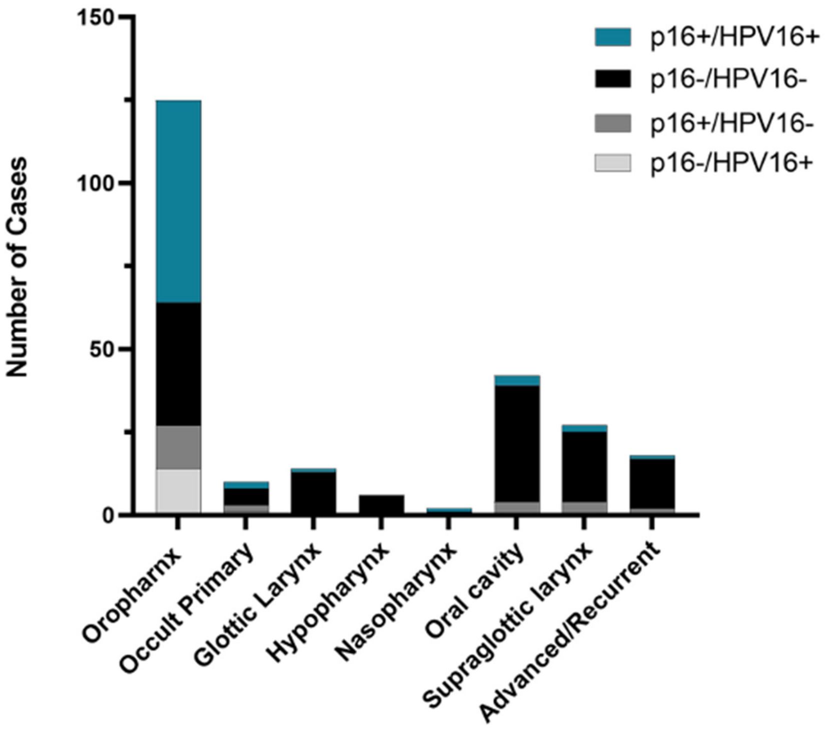

Figure S1. Site of origin of HNSCC in the study and p16/HPV discordance: Number of cases within molecular subtypes in HNSCC subgroups defined by primary tumor site and p16/HPV discordance.
